# A Proximal Sensor-Based Approach for Clean, Fast, and Accurate Assessment of the *Eucalyptus* spp. Nutritional Status and Differentiation of Clones

**DOI:** 10.3390/plants12030561

**Published:** 2023-01-26

**Authors:** Renata Andrade, Sérgio Henrique Godinho Silva, Lucas Benedet, Elias Frank de Araújo, Marco Aurélio Carbone Carneiro, Nilton Curi

**Affiliations:** 1 Department of Soil Science, Federal University of Lavras, Lavras 37200-900, MG, Brazil; 2 CMPC Celulose RioGrandense, Guaíba 92702-320, RS, Brazil

**Keywords:** portable X-ray fluorescence (pXRF) spectrometry, proximal sensing, machine learning, leaf nutrient analysis, greentech analysis, *Eucalyptus* cultivation, plant mineral nutrition

## Abstract

Several materials have been characterized using proximal sensors, but still incipient efforts have been driven to plant tissues. *Eucalyptus* spp. cultivation in Brazil covers approximately 7.47 million hectares, requiring faster methods to assess plant nutritional status. This study applies portable X-ray fluorescence (pXRF) spectrometry to (i) distinguish *Eucalyptus* clones using pre-processed pXRF data; and (ii) predict the contents of eleven nutrients in the leaves of *Eucalyptus* (B, Ca, Cu, Fe, K, Mg, Mn, N, P, S, and Zn) aiming to accelerate the diagnosis of nutrient deficiency. Nine hundred and twenty samples of *Eucalyptus* leaves were collected, oven-dried, ground, and analyzed using acid-digestion (conventional method) and using pXRF. Six machine learning algorithms were trained with 70% of pXRF data to model conventional results and the remaining 30% were used to validate the models using root mean square error (RMSE) and coefficient of determination (R^2^). The principal component analysis clearly distinguished developmental stages based on pXRF data. Nine nutrients were accurately predicted, including N (not detected using pXRF spectrometry). Results for B and Mg were less satisfactory. This method can substantially accelerate decision-making and reduce costs for *Eucalyptus* foliar analysis, constituting an ecofriendly approach which should be tested for other crops.

## 1. Introduction

As the most abundantly planted forest tree worldwide (over 20 million ha), *Eucalyptus* cultivation has been a prosperous economic activity. In Brazil, it accounts for 78% of the national forestry sector, worth over USD 21 billion in 2021 [1]. From cellulose and charcoal to timber and energy, many industry sectors depend on high-quality woody biomass provided by *Eucalyptus* planted forests [2]. In addition to being rich in essential oils (EO) [3], *Eucalyptus* has a great diversity of biological, pharmaceutical, and cosmetic applications [4]. Additionally, due to their phytotoxic and allelopathic properties, *Eucalyptus* leaves can be used as bioherbicide to suppress the growth of undesirable plant species, such as weeds [5,6].

*Eucalyptus* is widely used for forestation due to its resistance to diseases and easy adaptation to most different habitats [7]. Different species of the genus present fast growth and high yield, which makes them indispensable in the cellulose and paper industry, whose demand for raw materials is high [8]. In this sense, ensuring an adequate plant nutritional status is the key to guaranteeing high productivity with elevated quality.

Although soil is the essential matrix analyzed for the technical recommendation of fertilizers [9], foliar analysis reflects nutrient content actually taken up by plants, and it constitutes the best indicator of plant nutritional status [10]. However, due to the substantial number of steps involved in the process, the analytical standard method for foliar analysis is labor consumptive [11]. It requires sample pretreatment (oven-dried and ground leaf samples), expensive laboratory equipment, and hazardous chemicals. Another drawback of this conventional approach is the time elapsed from all analytical procedures, which diminishes the feasibility of analyzing many samples in short periods and in a specific stage of plant development. 

An alternative approach for foliar analysis is to reduce the number of steps in the analytical sequence and eliminate chemical waste generation. Currently, such requirements can be fulfilled using portable X-ray fluorescence (pXRF) spectrometry. Based on the fluorescence principle, this analysis allows quantitative measurement of several chemical elements (from Mg to U) in a fast (60 s) and cheap way [12]. When an atom is irradiated with energetic photons, an inner shell electron is ejected from its orbital. Thus, an outer shell electron moves to the inner shell to fill the void, but in doing so, characteristic (unique to each element) fluorescence energy is released [13]. pXRF spectrometry promotes non-destructive analyses that can be performed in field or laboratory conditions using a very small sample [14].

Despite all the advantages, this approach faces some limitations, such as the inability of pXRF to detect light elements (e.g., N and B) since such elements release low energy after they are reached by X-rays, preventing their detection by the equipment [12,13]. In addition, pXRF spectrometry generates a large dataset that may be difficult to handle. Thus, it is usually necessary to pre-process the elemental data to remove some inconsistent results, and a feature selection procedure may clarify which pXRF variables are the best to predict each target property. Moreover, the use of machine learning tools may accelerate the data processing [15,16] and also associate pXRF spectrometry reported numerical variables with categorical variables (e.g., classes of macro and micronutrients deficiency or surplus [16]).

Several studies have previously supported the applicability of this modern greentech approach for water [17], soil [18,19,20] (being already recognized as an official soil analysis method by the US Environmental Protection Agency [13,21]), and other vegetable matrices, such as needles, thatch, tree bark, deciduous leaves, and sapwood [22,23,24,25]. However, there are very few studies discussing pXRF spectrometry application for foliar analysis in the literature [26,27,28,29], and none of them focused specifically on *Eucalyptus*. So, this highly novel approach for foliar analysis remains sparsely explored.

This study aimed to apply pXRF spectrometry data to: (1) characterize the elemental chemical composition of *Eucalyptus* leaves in different development stages; (2) evaluate the feasibility of predicting *Eucalyptus* spp. nutritional status regarding macro (N, Ca, K, P, Mg, and S) and micronutrients (B, Cu, Fe, Mn, Zn) both numerically and per class of nutrient status (low, adequate, and high); and (3) distinguish different *Eucalyptus* spp. clones. We hypothesize that pXRF spectrometry in tandem with machine learning algorithms constitute a suitable alternative method for clean, fast, and accurate prediction of *Eucalyptus* spp. nutritional status and clone differentiation.

## 2. Results and Discussion

### 2.1. Elemental Chemical Characterization of Eucalyptus Leaves Using pXRF Spectrometry

The summary statistics of macro and micronutrients of *Eucalyptus* leaves for field and nursery plants is shown in Table 1. The results showed greater variability in the content of micronutrients (mainly Mn and Cu, CV% > 45.0), which was formerly registered [27]. The contents reported using the conventional method were greater than those reported using pXRF spectrometry (Table 2). Costa Junior et al. [26] also found greater contents when using conventional (wet-chemistry) analysis. Those authors attributed these differences to the irregular distribution of nutrients in the leaves.

Using the conventional method, some nutrients presented greater average contents for nursery plants (N, P, K, S, Cu, and Zn), while others presented greater average contents for field plants (Ca, Mg, B, Mn, and Fe) (Table 1). Such a pattern was also observed pXRF results (Table 2).

**Figure 1:**
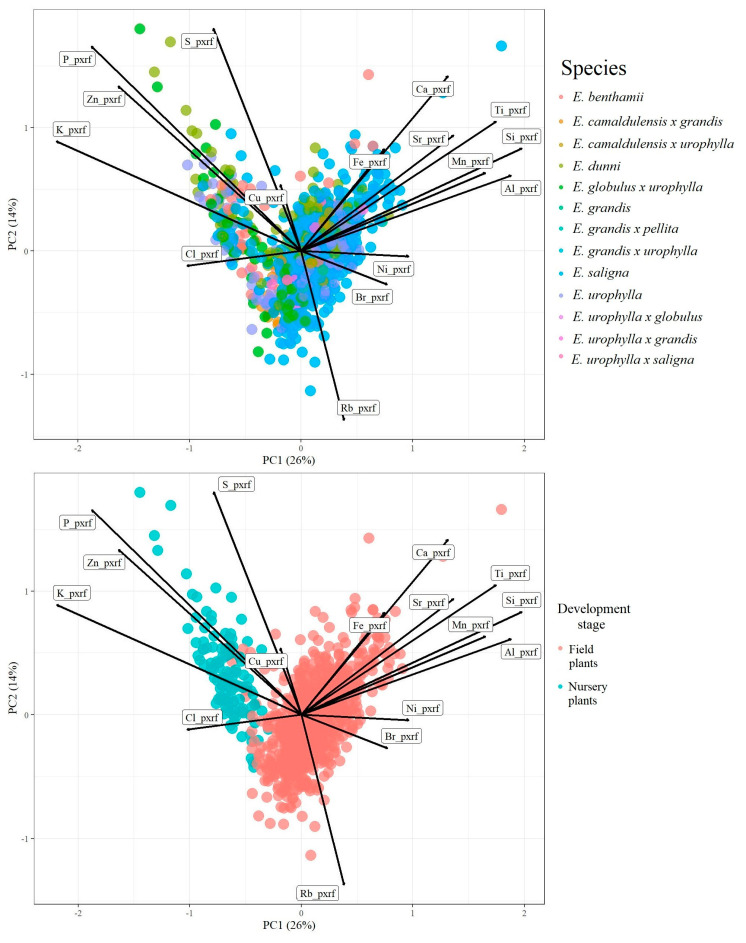
Principal component analysis (PCA) of portable X-ray fluorescence (pXRF) elemental data for *Eucalyptus* spp. leaves in different development stages.

Figure 1 shows the clear ability of pXRF data to identify differences in foliar nutritional contents in two development stages of *Eucalyptus.* Contrariwise, the thirteen *Eucalyptus* clones did not present any obvious visual pattern of distinction (Figure 1). Together, PC1 (26%) and PC2 (14%) explained 40% of the data variability. The greater values for P, Zn, K, and S elemental composition were found in nursery plants and were represented by the prominent black lines in this direction. Conversely, the prominence of Al, Ca, Mn, Fe, Si, Sr, and Ti black lines were found in the field plant direction, indicating that the great values of such elements were found in this developmental stage of the plants.

Greater average contents of Al, Fe, Ni, Sr, Ti, and V were found in leaves of field plants, probably reflecting inheritance from the parent material of soils [30]. As these plants were in direct contact with the soil matrix, the uptake of such elements by roots was expected [31].

### 2.2. Validation Performance of Leaf Nutrient Predictions

**Figure 2:**
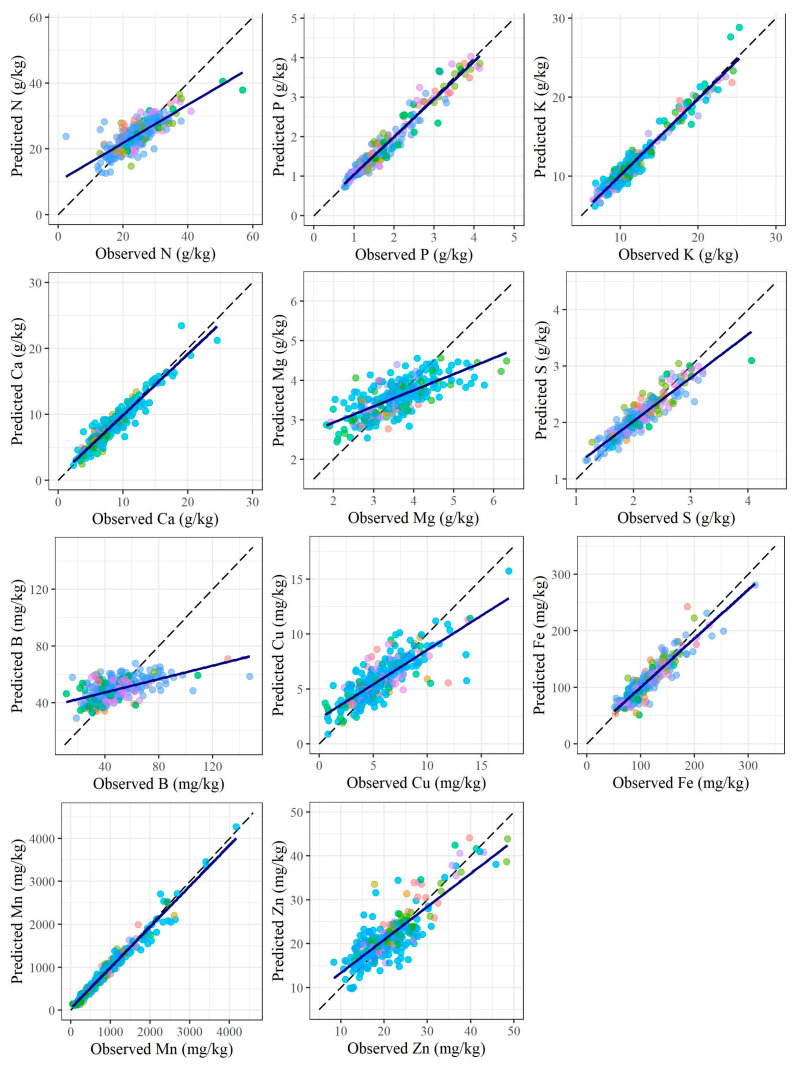
Observed versus predicted scatter plots for the best macro and micronutrients prediction models of *Eucalyptus* spp. leaves based on portable X-ray fluorescence (pXRF) elemental data.

According to the authors’ knowledge, the results described hereafter have not been reported in the literature previously. Outstanding outcomes were found using foliar macro and micronutrients prediction models, which are shown in Figure 2 and Table 3. The observed and predicted scatter plots highlight the adequacy of fit for almost all nutrients (N, P, K, Ca, S, Cu, Fe, Mn, and Zn) (Figure 2).

Despite the expected high variability between field and nursery foliar nutrient contents, the prediction models achieved elevated accuracy for both of them (Table 3). Remarkable results were found for P (R^2^ = 0.94, RPD = 4.13), K (0.95, 4.59), Ca (0.90, 3.17), S (0.85, 2.62), Fe (0.85, 2.54), Mn (0.97, 5.88), and Zn (0.75, 1.92). Reasonable results were achieved for N (R^2^ = 0.64, RPD = 1.66) and Cu (0.66, 1.69) (Table 3). Borges et al. [27] achieved variable results for macro (R^2^ ranging from 0.01 to 0.81) and micronutrients (from 0.77 to 0.99) prediction using pXRF data. Soares et. al. [29], who used pXRF data to predict phosphorus concentration in sugarcane leaves, also achieved good results, with R^2^ = 0.87. McGladdery et al. [24] used pXRF spectrometry to scan 228 organic material samples (thatch, deciduous leaves, grasses, tree bark, and herbaceous plants) and found poor results for Ca R^2^ = (0.11) and K (0.56), reasonable results for Mn (0.63) and Zn (0.61), and remarkable outcomes for Fe (0.80) and Cu (0.98). The literature has reported that the pXRF data alone have been sufficient for accurate predictions, which was confirmed for most macro and micronutrients evaluated in this work.

Nitrogen cannot be detected by pXRF spectrometry, but as it correlated well with sulfur detected by pXRF, it could be reasonably predicted (Figure 2, Table 3) (further discussions in the next sections). Nitrogen and sulfur are both essential components of catalysts and intermediates of primary metabolism, and both are found in amino acids, and hence in proteins, and nucleotides, and hence in nucleic acids [32]. These aspects should give meaning to the observed correlation, which is also expected to occur in other plant species (require further investigations). Neither previous studies attempted to use pXRF data to predict foliar N (R^2^ = 0.64) or B (R^2^ = 0.33) as here, nor did they assess nutrient status class predictions (as discussed further in the next section).

The boron and magnesium prediction models revealed the worst performances. Validation indices were 0.33 and 0.49 (R^2^), and 1.21 and 1.40 (RPD) for B and Mg, respectively. Boron also was not detected by pXRF, since it is a very light element [12], and none of the other pXRF variables correlated well with it. Although Mg was detected using pXRF spectrometry, it tended to produce very low fluorescence energy, which can be jeopardized using spectral influence (i.e., overlapping of peaks of different elements in the spectrum). Further studies should consider performing B and Mg scanning under vacuum conditions, as advised by Towett et al. [28].

The chemical composition of leaf nutrients determined by pXRF spectrometry was different from the leaf contents achieved using conventional laboratory analysis (Table 1 and Table 2), placing emphasis on the fundamental role of data modeling for accurate predictions of them. The best prediction models were achieved using the random forest (5 times), cubist regression (3 times), support vector machine (2 times), and projection pursuit regression (1 time) algorithms. The random forest algorithm has proven to be very robust for soil nutrient prediction [16,19,33], and also for other soil attributes such as particle size distribution [18] and fertility [20,34].

### 2.3. Validation of Leaf Nutrient Categorical Predictions

**Figure 3:**
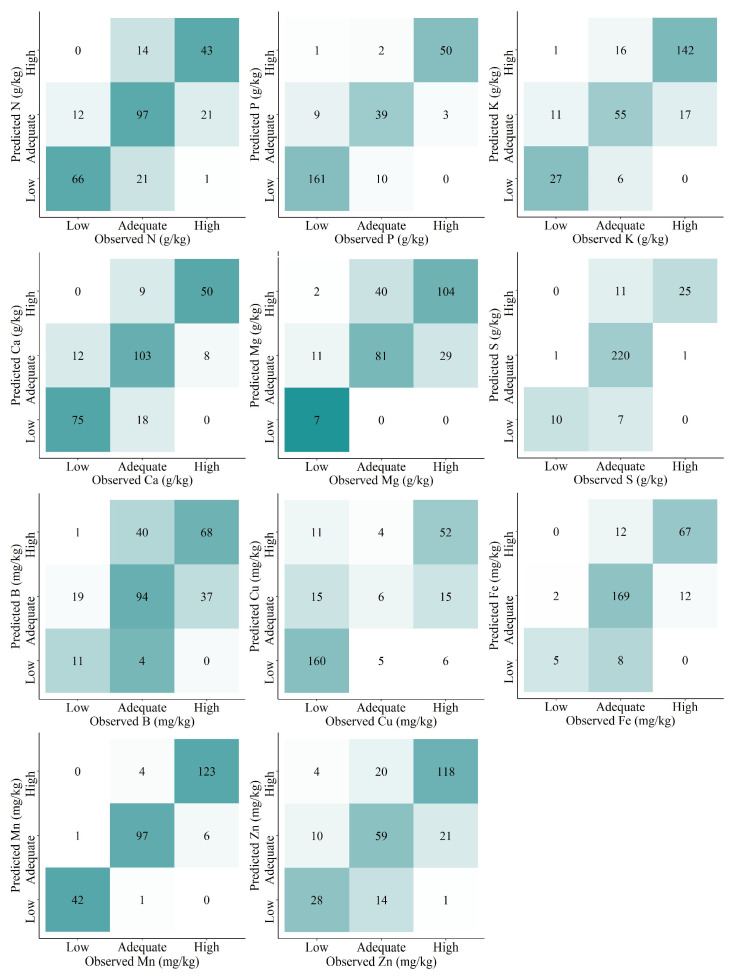
Confusion matrix for the best prediction models of *Eucalyptus* spp. leaves macro and micronutrients per class of nutrient status based on portable X-ray fluorescence (pXRF) elemental data. Darker colors indicate higher number of samples in that class.

The results found for predictions of foliar macro and micronutrients status classes are shown in Figure 3 and Table 4. Although even the small errors between observed versus predicted values were reflected in an increase in RMSE (Figure 2, Table 3), these errors were not always reflected in misclassifications of the nutrient status classes (categorical validation). This trend was corroborated by the high overall accuracies (number of correctly predicted samples) for all nutrients (from 0.63 to 0.96), even for B (0.63) and Mg (0.70). The Cohen’s Kappa coefficient values ranged from 0.35 to 0.93 (Table 4) and the correct and wrong predictions of classes of nutritional status can be better visualized in the confusion matrix (Figure 3).

The highest overall accuracies found for leaf macronutrients followed the order S > P > Ca > K > N > Mg, and for leaf micronutrients, Mn > Fe > Cu > Zn > B. The categorical validation showed the practical performance of the prediction models while considering the nutrient status classes. Classification models constitute an elegant solution to compensate for datasets with many outliers, or when only a mixed dataset (composed of both categorical and numerical information) is available [16,18]. In this sense, even B and Mg that obtained poor R^2^ values (0.33 and 0.49, respectively) could be reasonably and well predicted, respectively, using categorical modeling (B, overall accuracy = 0.63, and Mg, overall accuracy = 0.70).

**Figure 4:**
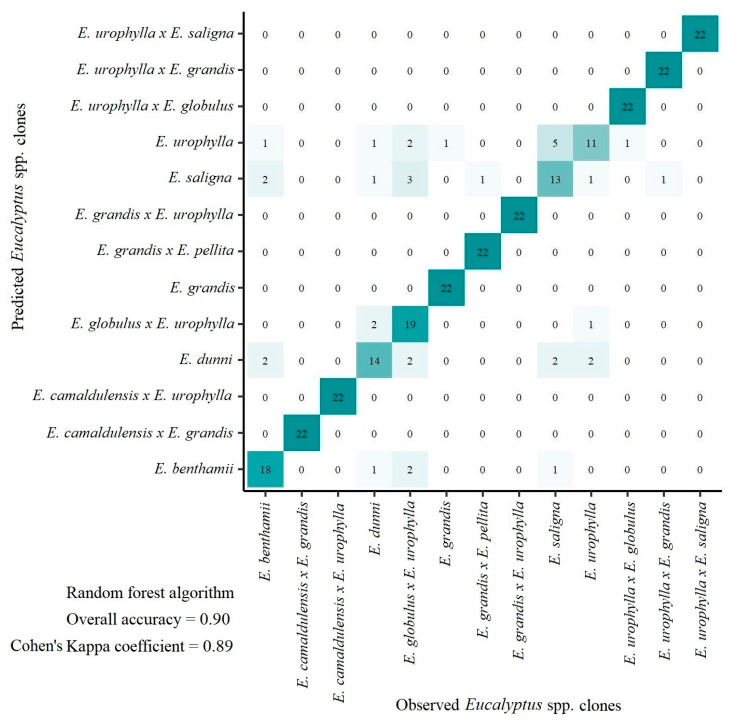
Confusion matrix of *Eucalyptus* spp. clone prediction models based on portable X-ray fluorescence (pXRF) elemental data. Darker colors indicate higher number of samples in that class.

The categorical prediction model used to distinguish *Eucalyptus* clones achieved remarkable high accuracy (overall accuracy = 0.90 and Cohen’s Kappa coefficient = 0.89) (Figure 4). These results demonstrate the promising potential of using pXRF data in tandem with machine learning algorithms to differentiate *Eucalyptus* clones. Although a visual differentiation pattern was not clearly observed in Figure 1, the random forest algorithm paved the way for distinguishing the thirteen *Eucalyptus* clones using foliar elemental chemical composition.

Knowing to which clone a specific sample belongs is a very useful information, for example, when sample identification is lost during field collection or when handling many samples at the same time in laboratory conditions. Furthermore, it is a rapid, cheap, and effective way to identify *Eucalyptus* clones in the field or at the seedling stage, especially because, in general, they are quite difficult to distinguish visually, mainly by non-experts. *E. urophylla*, *E. saligna,* and *E. dunni* were the most misclassified clones with 50%, 59%, and 64% accuracy, respectively.

The results reported in this study along with the literature data support the promising application of pXRF spectrometry to assess *Eucalyptus* nutritional status (both numerical and categorical). Auxiliary information from other proximal sensors data, such as VisNIR (spectral signature) and NixPro (color scanning), should be included in future studies as predictor variables to improve B and Mg numerical prediction models.

### 2.4. Variables Importance

**Figure 5:**
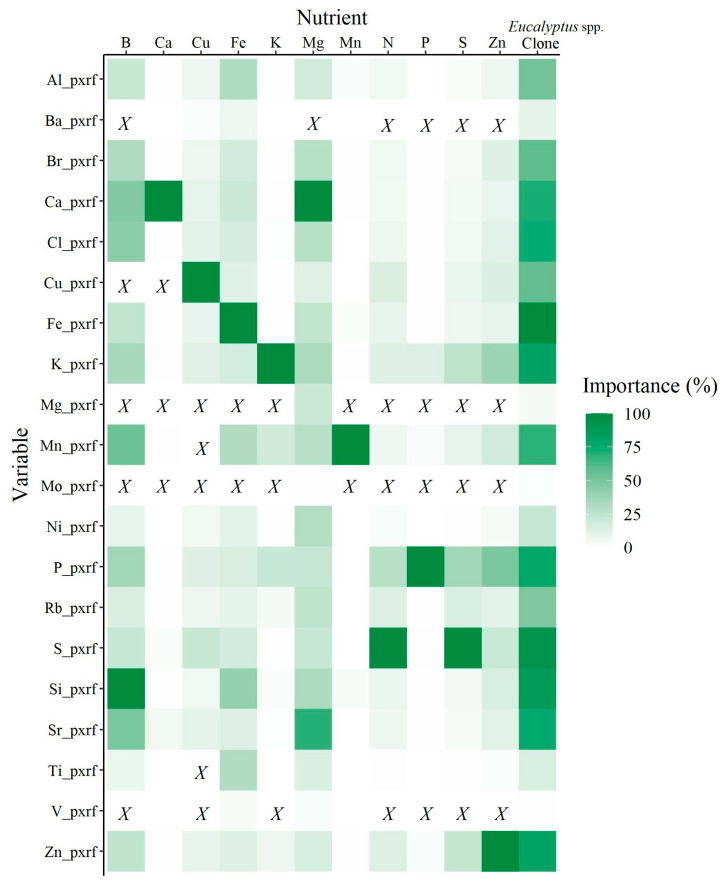
Explanatory variables used to predict the nutritional status of the *Eucalyptus* leaves and *Eucalyptus* spp. clones with their respective importance (%). *X* means that the variable was not used for the prediction model.

The most important variables to predict macro (N, P, K, Ca, Mg, and S) and micronutrients (B, Cu, Fe, Mn, and Zn) using pXRF data in *Eucalyptus* leaves are shown in Figure 5. The most important variables to predict P, K, Ca, S, Cu, Fe, Mn, and Zn were their respective elemental chemical composition acquired using pXRF spectrometry. Boron and nitrogen, which were not detected by pXRF spectrometry, had Si and S, respectively, as their most important explanatory variables (Figure 5). Magnesium and *Eucalyptus* spp. clone classification presented Ca and Fe as their most important variables.

In general, Mg and Mo (2 times each), V (5 times), Ba (6 times), Cu (10 times), Mn and Ti (11 times each) were the least important variables for the prediction models; all the remaining 13 pXRF variables were important for all prediction models (Figure 5). 

### 2.5. Data Variability versus Prediction Model Accuracy

**Figure 6:**
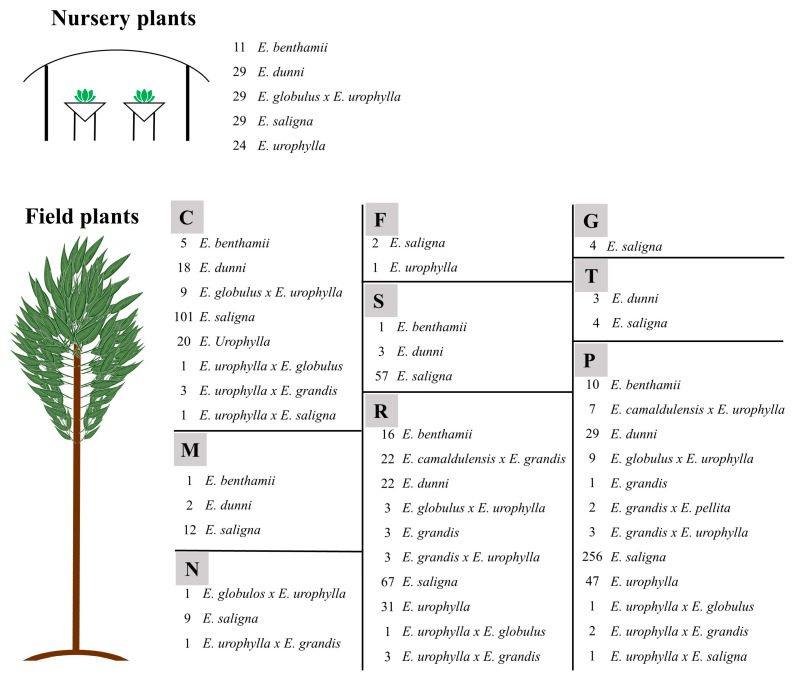
Overview of *Eucalyptus* spp. leaf collection and number of samples for each clone. C—Cambisol, F—Plintosol, G—Gleysol, M—Chernosol, N—Nitosol, P—Argisol, R—Neosol, S—Planosol, T—Luvosol.

Highly accurate prediction models for macro (N, P, K, Ca, Mg, and S) and micronutrients (B, Cu, Fe, Mn, and Zn) in *Eucalyptus* leaves have not been reported previously, especially with a large (920 samples) and diverse dataset. This study considered thirteen *Eucalyptus* clones (Figure 6) in two development stages: nursery and field plants (located in nine different soil classes).

Plants obtain most nutrients through root uptake from the soil solution. Thus, the water regime in the soil governs the processes of root interception, mass flow, and diffusion, since plant nutrition is impaired by water stress, which is reflected in the contents of nutrients in the leaves. The soil classes in which the *Eucalyptus* plants were planted vary highly in water retention and availability (Figure 6 and Table 5). However, this high variability did not decrease the prediction models’ accuracy (Figure 2 and Table 3).

Although some studies have shown that better results are achieved using a more homogeneous dataset, the prediction models for nutrients in *Eucalyptus* leaves may be extended to thirteen different *Eucalyptus* spp. clones. As the leaf is a matrix with less interference in the emission of fluorescence, we hypothesize that the differences in the nutritional composition of the *Eucalyptus* spp. clones do not interfere with the accuracy of the models. The different *Eucalyptus* spp. clones have differences in growth speed and in nutritional requirements so, at the same stage of development, a clone can have a greater or lesser content of a given nutrient. However, this was not a problem for the prediction models to navigate.

Different development stages of *Eucalyptus* spp. require different contents for each nutrient. As previously demonstrated, field and nursery plants have different nutritional compositions (Table 1). However, the generation of specific models for each development stage was not necessary.

Despite all the variability in the dataset, general prediction models (i.e., applicable to multiple conditions and with data from different clones and plant development stages) with high accuracy may be yielded (Figure 2, Figure 3 and Figure 4, and Table 3 and Table 4). Usually, general prediction models are not restricted by the number of samples [18,19,20], and can be extended to many different conditions.

### 2.6. Future Applications

Knowing the nutritional status of *Eucalyptus* spp. is fundamental for assuring high productivity and raw material security [35]. Well-nourished plants are more resilient to pests and diseases [36,37] and to hostile environmental conditions (e.g., drought season, water stress, etc.). Foliar analysis allows one to determine if plants are well-nourished or not. Determination of the contents of macro and micronutrients in the leaves, as they better reflect the nutritional status of the plant, allows one to identify deficiency or toxicity conditions [38,39,40]. In addition to being time-consuming, foliar analysis is also expensive.

In this sense, pXRF data in tandem with machine learning algorithms can be truly helpful in predicting *Eucalyptus* spp. nutritional status (both numerical and categorical), being complementary information used to select which fertilizers are most required and which are the best doses to recommend. In addition, it may confirm an observed visual deficiency of a given plant nutrient and can be used for fast temporal monitoring of the nutritional status, mainly for nursery plants, without increasing *Eucalyptus* spp. production costs. This greentech approach may be an accurate tool to distinguish symptoms caused by pathogenic agents [41,42,43] from those caused by inadequate nutrition in a very rapid way.

The feasibility of analyzing many leaf samples without generating chemical waste and in a specific stage of plant development, mainly for nursery plants, is another advantage of using pXRF spectrometry for foliar analysis. As the required contents of nutrients vary according to the plant development stage, this proximal sensor may be used routinely for leaf macro and micronutrient quantification. The generated models may be further improved with annually collected data, mainly for field plants, during the *Eucalyptus* spp. cycle (6 years for cellulose production), incorporating the concept of validity time to them.

Further research concerning this novel approach encompassing other *Eucalyptus* clones (there are more than 700 clones [44]) and other species of economic and environmental interest are highly encouraged.

## 3. Materials and Methods

### 3.1. Eucalyptus Leaves Sampling

*Eucalyptus* spp. leaves were collected at Rio Grande do Sul state, Brazil. In total, 920 leaf samples were collected encompassing 13 different clones and 2 different development stages: nursery and field (Figure 6). The field plants were distributed in 9 soil classes which were classified according to the Brazilian Soil Classification System [45] and US Soil Taxonomy [46] (Table 5).

### 3.2. Conventional Analysis of Eucalyptus Leaves

In the laboratory, the leaf samples were carefully washed with distilled water, oven-dried (60 °C) until constant weight, and ground (30 mesh). For the determination of N, Ca, Mg, P, K, S, B, Cu, Fe, Mn, and Zn the ground leaf material was wet-digested using the nitro-perchloric acid method [47,48]. The samples (0.5 g) were transferred to 50 mL glass digestion tubes, treated with 6 mL of acid solution (HNO_3_:HClO_4_ 2:1 *v*/*v*, purity grade 65 and 70, respectively, Sigma Aldrich, Schnelldorf, Germany), and digested using a heating block digestion system. After cooling, the final solutions were transferred to 50 mL volumetric flasks, and the volume was completed with ultrapure water. The resulting solutions were analyzed using inductively coupled plasma optical emission spectroscopy (ICP-OES) (Spectro Analytical Instruments Inc., Kleve, Germany) for nutrient quantification.

### 3.3. pXRF Spectrometry Analysis

A pXRF spectrometer (model Tracer 5g, Bruker Analytical Instrumentation, Billerica, MA, USA) was used to scan the samples and assess their elemental composition [49]. Scans were performed on the ground samples, in triplicate, in Plants mode for 60 s using the inbuilt Geochem software. In total, the pXRF spectrometer detected the concentration of twenty elements in all soil samples: Al, Ba, Br, Ca, Cl, Cu, Fe, K, Mg, Mn, Mo, Ni, P, Rb, S, Si, Sr, Ti, V, and Zn. This detection and quantification of elements was automatically completed using a pre-processed curve contained in the equipment rather than using the raw XRF spectrum.

In order to guarantee the quality of the data generated using the pXRF spectrometer, manufacturer’s standard check samples (CS-M2 and CS-P) had their contents acquired using pXRF and contrasted to certified reference values. From these certified materials, the recovery values obtained per element using the equipment were calculated (recovery value = elemental content obtained using pXRF/certified elemental content). The elements obtained using the pXRF analyses and their recovery values (CS-M2/CS-P) were: Al (–/–), Ba (3.48/–), Br (–/–), Ca (0.35/0.93), Cl (–/–), Cu (0.65/–), Fe (0.86/–), K (0.48/0.95), Mg (–/–), Mn (2.18/0.99), Mo (–/–), Ni (–/–), P (0.78/0.95), Rb (–/0.98), S (–/0.95), Si (–/–), Sr (1.08/–), Ti (–/–), V (–/–), and Zn (1.25/0.99). Dashes (–) indicate either the element has no certified content in the reference material, or it was not detected using the pXRF spectrometer.

### 3.4. Statistical Analyses and Modeling

Prior to the modeling, boxplots and a principal component analysis (PCA) were performed in order to remove outliers and better characterize the dataset. Afterwards, the *Eucalyptus* leaves dataset was randomly separated into modeling and validation sub-datasets, consisting of 70% (*n* = 644) and 30% (*n* = 276) of the total samples, respectively. For each nutrient, recursive feature elimination was used to select the optimal pXRF explanatory variables using the “boruta” package [50]. The method performs a top-down search for relevant features and ranks their importance. Thus, the pXRF variables were classified as Confirmed (important feature), Tentative (the feature does not impact model’s accuracy) or Rejected (not important feature). The pXRF variables selected for each nutrient and *Eucalyptus* spp. clone prediction models can be seen in Figure 5.

The prediction models were created using *K*-fold cross-validation (*K* = 10) using six different machine learning algorithms: projection pursuit regression (PPR), partial least squares (PLS), random forest (RF), support vector machine (SVM), extreme gradient boosting (XGB), and cubist regression. All the models were built in R software (version 4.2.1) [51] using the “caret” package [52].

**Figure 7:**
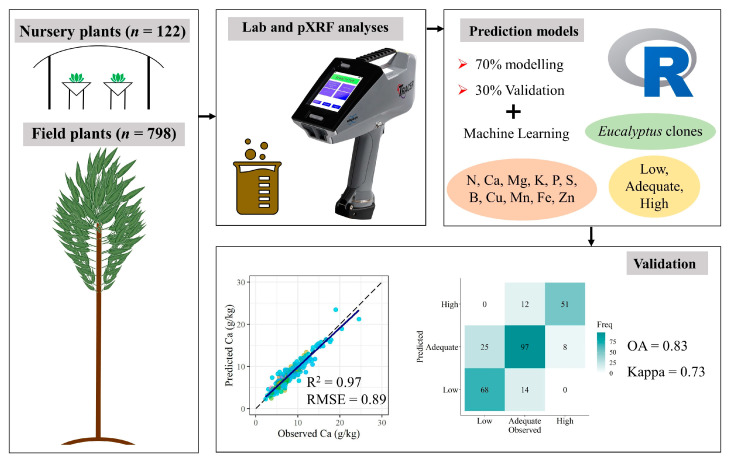
Flowchart illustrating the sequence of the methodological process of this work for predicting the nutrient status of *Eucalyptus* spp. leaves and *Eucalyptus* spp. clones.

The same data modeling process was performed for categorical predictions of *Eucalyptus* spp. clones and nutrient status classes using six different machine learning classification algorithms: C5.0, partial least squares (PLS), random forest (RF), support vector machine (SVM), extreme gradient boosting (XGB), and bagged cart (BC). As there was a high discrepancy in the number of samples of *Eucalyptus* spp. clones (e.g., *E. saligna* = 541, *E. benthamii* = 44), the dataset was balanced with upsampling and downsampling using the “groupdata2” package. Upsampling is performed with replacement for added rows, while the original data remain intact, and downsampling is performed without replacement, meaning that rows are not duplicated, but only removed [53]. A flowchart was constructed to facilitate the understanding of the complete methodological process implemented in this work (Figure 7).

### 3.5. Evaluation of Models’ Performance

The accuracy of the prediction models was evaluated by comparing the predicted with the observed values using the coefficient of determination (R^2^), root mean square error (RMSE) (Equation (1)), and residual prediction deviation (RPD) (Equation (2)). The equations are represented as:

(1) $$RMSE=\sqrt{\frac{1}{n}\sum_{i=1}^{n}{\left(y_{i}-m_{i}\right)}^{2}}$$

(2) RPD=SD/RMSE

where *n*: number of observations, *yi*: estimated value using the model, *mi*: measured value using the conventional chemical analysis, and *SD*: standard deviation of the observed values. The RPD values have been framed into three classes: RPD > 2, prediction models delivering accurate results; 1.4 ≤ RPD ≤ 2, prediction models providing moderately accurate results; and RPD < 1.4, prediction models being non-reliable [54]. The models with greater R^2^ and RPD and smaller RMSE values were considered optimal for predicting leaf conventional laboratory analysis.

The validation of predicted nutritional status (low, adequate, and high) and *Eucalyptus* spp. clones classification was performed using overall accuracy (ranging from 0 to 1, [55]) and Cohen’s Kappa coefficient (ranging from −1 to 1, [56]), calculated using Equations (3) and (4), respectively, in a confusion matrix as:

(3) $$Overall\;Accuracy=\frac{Pc}{N}$$

(4) $${Cohen}^{’}s\;Kappa=\frac{Po-Pe}{1-Pe}$$

where *Pc* is the sum of the confusion matrix’s main diagonal (predicted nutritional status classes), *N* is the total number of validation samples, *Po* is the observed agreement, and *Pe* is the probability of random agreement [57]. The models with greater overall accuracy and Cohen’s Kappa coefficient were considered the best ones to predict *Eucalyptus* spp. clones and nutritional status.

## 4. Conclusions

This study is the first attempt to use pXRF data to predict macro and micronutrients contents in *Eucalyptus* spp. leaves. The elemental chemical characterization obtained using this proximal sensor may fairly reflect conventional (wet-chemistry) laboratory analysis. The results demonstrated the promising potential of using pXRF spectrometry for numerical and categorical predictions of N, P, K, Ca, S, Cu, Fe, Mn, and Zn (R^2^ ranging from 0.64 to 0.97), and categorical prediction of B and Mg (overall accuracy of 0.63 and 0.70, respectively), in addition to being able to differentiate thirteen *Eucalyptus* spp. clones (overall accuracy = 0.90). 

Our findings may pave the way for further studies into plant clone differentiation and rapid assessment of their nutritional status using a cheap, fast, and environmentally friendly method. Further studies are encouraged to test this modern foliar nutrient analysis on other plant species, representing alternatives to reduce costs and time needed for assessing such data, supporting agronomic and environmental strategies.

## Figures and Tables

**Table 1:** Descriptive statistics of macro (g kg^−1^) and micronutrients (mg kg^−1^) using conventional analysis of *Eucalyptus* spp. leaves for field and nursery plants, separately and combined (total value).

Nutrient	Dataset	Min	Max	Mean	STD ^1^	CV (%) ^2^
N	Total	0.00	56.82	23.85	6.19	25.95
	Field	0.00	37.84	22.99	5.54	24.12
	Nursery	2.36	56.82	29.52	7.16	24.25
P	Total	0.62	5.14	1.64	0.70	42.74
	Field	0.62	3.40	1.43	0.39	27.60
	Nursery	1.83	5.14	3.07	0.61	19.83
K	Total	5.54	25.60	11.56	3.67	31.77
	Field	5.54	24.36	10.41	2.12	20.34
	Nursery	12.67	25.60	19.11	2.60	13.61
Ca	Total	2.32	29.04	8.19	3.29	40.23
	Field	2.32	29.04	8.27	3.48	42.02
	Nursery	3.52	13.18	7.63	1.57	20.52
Mg	Total	1.57	7.10	3.55	0.74	20.94
	Field	1.57	7.10	3.58	0.77	21.45
	Nursery	2.23	4.64	3.32	0.49	14.89
S	Total	0.98	4.06	2.10	0.41	19.79
	Field	0.98	3.30	2.01	0.34	17.07
	Nursery	1.73	4.06	2.65	0.42	15.85
B	Total	11.24	147.50	48.14	18.27	37.95
	Field	11.24	147.50	49.48	18.87	38.14
	Nursery	21.82	79.74	39.33	9.90	25.18
Mn	Total	29.74	4170.40	855.12	683.10	79.88
	Field	62.22	4170.40	965.95	666.88	69.04
	Nursery	29.74	498.00	130.19	61.93	47.57
Fe	Total	40.30	344.10	110.85	38.03	34.31
	Field	40.30	344.10	114.39	38.22	33.42
	Nursery	53.13	224.90	87.72	27.21	31.01
Cu	Total	0.07	27.41	5.65	2.71	47.96
	Field	0.07	17.58	5.59	2.53	45.20
	Nursery	1.64	27.41	6.03	3.67	60.94
Zn	Total	8.30	89.63	21.73	8.18	37.64
	Field	8.30	89.63	19.86	5.48	27.59
	Nursery	17.17	80.20	33.91	11.73	34.58

^1^ Standard deviation; ^2^ Coefficient of variation.

**Table 2:** Descriptive statistics of portable X-ray fluorescence (pXRF) elemental data (%) of *Eucalyptus* spp. leaves for nursery and field plants, separately and combined (total value).

Statistics	Al	Ba	Br *	Ca	Cl	Cu *	Fe	K	Mg	Mn	Mo *	Ni *	P	Rb *	S	Si	Sr *	Ti *	V *	Zn
	Total																			
Min	0.04	0.01	0.02	0.53	0.02	0.03	0.00	0.31	0.20	0.00	0.02	0.01	0.03	0.02	0.01	0.01	0.04	0.03	0.01	0.00
Max	0.28	0.03	3.60	3.56	0.80	0.22	0.10	2.36	0.53	0.85	0.04	0.53	0.38	0.76	0.33	0.59	5.27	0.39	0.03	0.01
Mean	0.08	0.01	0.43	1.10	0.29	0.08	0.01	0.81	0.26	0.13	0.03	0.03	0.10	0.13	0.12	0.09	0.45	0.05	0.01	0.00
STD ^1^	0.03	0.00	0.42	0.33	0.13	0.02	0.01	0.32	0.05	0.12	0.01	0.03	0.04	0.09	0.03	0.06	0.37	0.03	0.00	0.00
CV (%) ^2^	32.24	32.07	98.38	29.77	45.25	28.94	54.38	38.98	20.94	86.64	21.71	96.50	35.46	70.91	23.65	61.47	81.77	50.35	24.74	41.15
	Field																			
Min	0.04	0.01	0.02	0.53	0.02	0.03	0.00	0.31	0.20	0.01	0.02	0.01	0.03	0.03	0.01	0.01	0.04	0.03	0.01	0.00
Max	0.28	0.03	3.60	3.56	0.80	0.22	0.10	1.80	0.53	0.85	0.04	0.53	0.20	0.76	0.33	0.59	5.27	0.39	0.03	0.01
Mean	0.09	0.01	0.48	1.11	0.28	0.08	0.01	0.71	0.26	0.15	0.03	0.03	0.09	0.13	0.12	0.10	0.48	0.05	0.01	0.00
STD ^1^	0.03	0.00	0.43	0.34	0.13	0.02	0.01	0.19	0.06	0.11	0.01	0.03	0.02	0.09	0.03	0.06	0.38	0.03	0.00	0.00
CV (%) ^2^	32.00	32.00	91.18	30.87	46.98	28.92	54.95	25.95	21.31	75.35	23.22	96.42	23.47	69.16	23.06	56.68	79.47	50.35	24.74	27.34
	Nursery																			
Min	0.05	0.01	0.04	0.55	0.16	0.05	0.01	0.85	0.20	0.00	0.03	0.00	0.09	0.02	0.08	0.01	0.08	0.00	0.00	0.00
Max	0.11	0.01	0.28	1.65	0.74	0.21	0.03	2.36	0.33	0.08	0.04	0.00	0.38	0.06	0.28	0.29	0.63	0.00	0.00	0.01
Mean	0.07	0.01	0.12	1.01	0.39	0.08	0.01	1.45	0.24	0.02	0.03	0.00	0.17	0.04	0.15	0.04	0.24	0.00	0.00	0.00
STD ^1^	0.02	0.00	0.05	0.17	0.10	0.02	0.00	0.25	0.04	0.01	0.00	0.00	0.04	0.01	0.03	0.03	0.10	0.00	0.00	0.00
CV (%) ^2^	23.25	12.79	44.11	16.75	26.50	29.17	33.05	17.39	17.73	66.13	14.81	0.00	23.83	28.51	19.81	89.18	42.12	0.00	0.00	40.47

^1^ Standard deviation; ^2^ Coefficient of variation; * Values multiplied by 100 for better visualization of data.

**Table 3:** Root mean square error (RMSE), coefficient of determination (R^2^), and residual prediction deviation (RPD) for macro and micronutrients prediction models of *Eucalyptus* spp. leaves based on portable X-ray fluorescence (pXRF) elemental data.

Nutrient	Model	RMSE	R^2^	RPD	Nutrient	Model	RMSE	R^2^	RPD
N	PPR	4.46	0.51	1.42	B	PPR	15.48	0.26	1.16
	PLS	4.56	0.49	1.39		PLS	15.63	0.24	1.15
	RF	**3.81**	**0.64**	**1.66**		RF	**14.82**	**0.33**	**1.21**
	SVM	4.49	0.51	1.41		SVM	15.65	0.26	1.15
	XGB	4.28	0.55	1.48		XGB	16.46	0.21	1.09
	Cubist	3.97	0.62	1.60		Cubist	15.47	0.26	1.16
P	PPR	0.19	0.94	3.94	Cu	PPR	1.59	0.63	1.62
	PLS	0.19	0.93	3.85		PLS	1.57	0.64	1.64
	RF	**0.18**	**0.94**	**4.13**		RF	1.70	0.57	1.51
	SVM	0.20	0.93	3.81		SVM	**1.52**	**0.66**	**1.69**
	XGB	0.24	0.90	3.12		XGB	1.97	0.45	1.31
	Cubist	0.20	0.93	3.72		Cubist	1.64	0.60	1.57
K	PPR	0.89	0.95	4.40	Fe	PPR	16.04	0.81	2.31
	PLS	0.93	0.94	4.22		PLS	17.40	0.80	2.13
	RF	0.92	0.95	4.25		RF	19.39	0.78	1.91
	SVM	0.92	0.94	4.24		SVM	**14.58**	**0.85**	**2.54**
	XGB	0.90	0.95	4.35		XGB	19.21	0.74	1.93
	Cubist	**0.86**	**0.95**	**4.59**		Cubist	15.61	0.83	2.37
Ca	PPR	1.05	0.89	3.05	Mn	PPR	**113.00**	**0.97**	**5.88**
	PLS	1.06	0.89	3.02		PLS	132.00	0.96	5.04
	RF	**1.01**	**0.90**	**3.17**		RF	129.34	0.96	5.14
	SVM	1.04	0.90	3.08		SVM	128.51	0.96	5.17
	XGB	1.11	0.88	2.89		XGB	154.38	0.95	4.31
	Cubist	1.03	0.90	3.10		Cubist	123.07	0.97	5.40
Mg	PPR	0.59	0.40	1.29	Zn	PPR	4.67	0.67	1.73
	PLS	0.63	0.31	1.20		PLS	4.82	0.64	1.68
	RF	0.55	0.49	1.39		RF	**4.22**	**0.75**	**1.92**
	SVM	0.64	0.30	1.19		SVM	4.84	0.65	1.67
	XGB	0.61	0.36	1.25		XGB	4.27	0.72	1.90
	Cubist	**0.55**	**0.49**	**1.40**		Cubist	4.56	0.69	1.77
S	PPR	0.18	0.81	2.27					
	PLS	0.19	0.79	2.17					
	RF	0.17	0.85	2.52					
	SVM	0.18	0.82	2.34					
	XGB	0.20	0.78	2.05					
	Cubist	**0.16**	**0.85**	**2.62**					

PPR: projection pursuit regression; PLS: partial least squares; RF: random forest; SVM: support vector machine; and XGB: extreme gradient boosting. The optimal results obtained for each nutrient are given in bold.

**Table 4:** Overall accuracy (OA) and Cohen’s Kappa coefficient (CKC) for macro and micronutrients prediction models of *Eucalyptus* spp. leaves based on portable X-ray fluorescence (pXRF) elemental data.

Nutrient	Model	OA	CKC	Nutrient	Model	OA	CKC
N	C5.0	0.67	0.50	B	C5.0	0.63	0.33
	PLS	0.64	0.43		PLS	0.57	0.20
	RF	**0.75**	**0.60**		RF	0.61	0.30
	SVM	0.70	0.53		SVM	0.59	0.23
	XGB	0.72	0.56		XGB	0.62	0.32
	BC	0.73	0.57		BC	**0.63**	**0.35**
P	C5.0	0.91	0.83	Cu	C5.0	0.80	0.59
	PLS	0.79	0.53		PLS	0.65	0.13
	RF	**0.91**	**0.83**		RF	**0.80**	**0.59**
	SVM	0.88	0.77		SVM	0.80	0.56
	XGB	0.89	0.80		XGB	0.79	0.58
	BC	0.89	0.81		BC	0.76	0.54
K	C5.0	0.80	0.64	Fe	C5.0	0.87	0.72
	PLS	0.68	0.38		PLS	0.85	0.64
	RF	0.80	0.65		RF	**0.88**	**0.73**
	SVM	0.80	0.64		SVM	0.88	0.71
	XGB	**0.81**	**0.67**		XGB	0.87	0.69
	BC	0.79	0.63		BC	0.87	0.72
Ca	C5.0	0.80	0.68	Mn	C5.0	0.94	0.90
	PLS	0.73	0.58		PLS	0.88	0.80
	RF	**0.83**	**0.73**		RF	0.94	0.91
	SVM	0.83	0.73		SVM	0.95	0.92
	XGB	0.80	0.68		XGB	**0.96**	**0.93**
	BC	0.79	0.66		BC	0.94	0.91
Mg	C5.0	0.62	0.32	Zn	C5.0	0.69	0.49
	PLS	0.67	0.38		PLS	0.67	0.41
	RF	**0.70**	**0.45**		RF	0.72	0.52
	SVM	0.65	0.35		SVM	**0.75**	**0.58**
	XGB	0.68	0.40		XGB	0.68	0.48
	BC	0.65	0.38		BC	0.71	0.51
S	C5.0	**0.93**	**0.75**				
	PLS	0.85	0.34				
	RF	0.91	0.67				
	SVM	0.91	0.70				
	XGB	0.92	0.69				
	BC	0.91	0.68				

PLS: partial least squares; RF: random forest; SVM: support vector machine; XGB: extreme gradient boosting; and BC: bagged cart. The optimal results obtained for each nutrient are given in bold.

**Table 5:** Soil classes where the field plants were distributed, according to the Brazilian System of Soil Classification (SiBCS) and US Soil Taxonomy.

Identification of Figure 6	SiBCS	Soil Taxonomy
C	Cambisol	Inceptisol
F	Plinthosol	Oxisol
G	Gleysol	Entisol (Aquent)
M	Chernosol	Mollisol
N	Nitosol	Ultisol
P	Argisol	Ultisol
R	Neosol	Entisol
S	Planosol	Alfisol
T	Luvisol	Alfisol

## Data Availability

The data presented in this study are available on request from the corresponding author. The data are not publicly available due to the very large amount of data.
